# Spatial Effects of Permethrin-Impregnated Bed Nets on Child Mortality: 26 Years on, a Spatial Reanalysis of a Cluster Randomized Trial

**DOI:** 10.4269/ajtmh.19-0111

**Published:** 2019-10-07

**Authors:** Christopher I. Jarvis, Lea Multerer, Daniel Lewis, Fred Binka, W. John Edmunds, Neal Alexander, Thomas A. Smith

**Affiliations:** 1 London School of Hygiene and Tropical Medicine, London, United Kingdom;; 2 MRC London Hub for Trials Methodology Research, London, United Kingdom;; 3 Department of Epidemiology and Public Health, Swiss Tropical and Public Health Institute, Basel, Switzerland;; 4 University of Basel, Basel, Switzerland;; 5 School of Public Health, University of Health and Allied Sciences, Ho, Ghana

## Abstract

In addition to the direct effect of insecticide-treated nets (ITNs), there has been evidence for spatial indirect effects. Spatial analyses in cluster randomized trials (CRTs) are rare, but a large-scale CRT from 1993 was one of the first to conduct a spatial analysis of ITNs in CRTs. We revisit these data by applying a broader range of contemporary spatial methods to further explore spatial spillover. We conducted three analyses: 1) exploratory spatial analysis, considering spatial patterns and spillover in the data; 2) spatial modeling, estimating the intervention effect considering spatial effects; and 3) analysis of distance-based spillover and interaction with the intervention, characterizing the functional distance over which the spillover effect was present. There were consistent indications of spatial patterns from the exploratory analysis. Bed nets were associated with a 17% reduction in all-cause mortality for children aged 6–59 months, and the intervention estimate remained robust when allowing for the spatial structure of the data. There was strong evidence of a spatial spillover effect: for every additional 100 m that a control household was from an intervention household (and vice versa), the standardized mortality ratio (SMR) increased by 1.7% (SMR 1.017, 95% credible interval 1.006–1.026). Despite evidence of a spatial spillover effect, the conclusions of the trial remain unaffected by spatial model specifications. Use of ITNs was clearly beneficial for individuals, and there was compelling evidence that they provide an indirect benefit to individuals living nearby. This article demonstrates the extra utility that spatial methods can provide when analyzing a CRT.

## INTRODUCTION

A series of cluster randomized trials (CRTs) carried out two decades ago in endemic areas in Africa demonstrated strong evidence that insecticide-treated bed nets (ITNs) can reduce child mortality.^1–5^ For instance, a large-scale CRT of ITNs in the Kassena-Nankana (Navrongo) district of northern Ghana found a 17% reduction in all-cause child mortality in children aged 6 months to 4 years (standardized mortality ratio [SMR] 0.83, 95% CI: 0.69–1.00).^3^ The CRT began in July 1993 and provided 31,000 ITNs to intervention participants in 48 geographically defined polygon clusters. From a meta-analysis of all the CRTs involving ITNs, the average reduction in all-cause mortality was estimated to be 18% (RR: 0.82, 95% CI: 0.76–0.89).^6^

In addition to the direct effect, there was evidence for positive spillovers, or spatial indirect effects.^6–8^ Using data from the Navrongo CRT, a subsequent study by Binka et al.^9^ found reductions in mortality among individuals without ITNs who lived close to individuals who did. From a recent systematic review, this example of positive spillover was found to be the first evidence of a spatial indirect effect of the bed net intervention, in addition to the direct effect that ITNs had in reducing mortality for those using them.^10^ Binka et al. used information on household location to gain insight into spatial indirect effects. Subsequent analyses have also demonstrated positive spillover with ITNs,^5,7,8,11,12^ although at least one study failed to find evidence of spatial indirect effects.^13^

The approach to estimating the spatial indirect effect used by Binka et al.^9^ was novel at the time, but in subsequent years, the emergence of a subdisciplinary focus on spatial epidemiology has led to a broader range of applicable methods.^14,15^ The specification of new spatial models, particularly in light of the continued growth of computational capacity, and refinement of optimization methods have made advanced spatial regression approaches tractable. In this article, we revisit the Kassena-Nankana CRT using contemporary spatial methods to explore the existence of positive spillovers, estimate the spatial indirect effects, and consider their impact, if any, on the overall trial conclusions.

To the authors’ knowledge, this article presents the first time that many of these methods have been applied to a CRT. In addition, we propose a new method called cluster reallocation, which allows trialists to consider if spatial spillover is present in a CRT. As well as demonstrating the application of contemporary spatial methods, this enhanced and extended reanalysis demonstrates the additional utility of collecting global positioning system (GPS) coordinates during trials. We argue that beyond being a useful resource for trial management or for mapping trial context, an explicit analysis of location can yield important additional information about the functioning of particular interventions in CRTs.

## MATERIALS AND METHODS

### Study.

**Figure 1.**
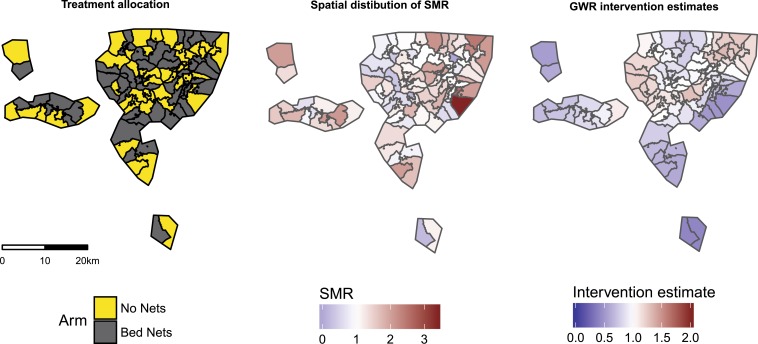
Study area, spatial variation of standardized mortality ratio, and spatial heterogeneity of intervention effect from the geographically weighted regression model. This figure appears in color at www.ajtmh.org.

The trial was conducted between July 1993 and June 1995 in the Kassena-Nankana district in the Upper East Region of Ghana. The study design has been described previously.^3^ In short, a parallel CRT with 96 geographically contiguous clusters (Figure 1) with an average of roughly 1,400 people per cluster and an average of 124 compounds per cluster was conducted. The intervention of permethrin-impregnated bed nets was allocated to 48 clusters and the outcome was all-cause mortality in children aged 6 months to 4 years.

### Data.

The data contain one record per compound, with variables for the location coordinates (Westings and Northings projected in WGS 84/Universal Transverse Mercator [UTM] zone 30 N), and the observed and expected number of deaths per compound. As per Binka et al.,^9^ “The expected number of deaths for each cluster was calculated by applying age-specific death rates derived from preintervention population to the postintervention time at risk.” The distance from intervention households to the nearest control and vice versa, referred to as the distance to the discordant pair, had been calculated previously.^9^ These distances were verified by calculating the Euclidean distance based on the UTM coordinates in QGIS 2.18.14^16^ and R 3.4.2.^17^ The outcome is an SMR and was calculated by dividing the observed deaths in each household by the expected number of deaths.

### Statistical analyses.

We conducted three broad sets of analyses: 1) exploratory spatial analysis, considering spatial patterns and spillover in the data; 2) spatial modeling, estimating the intervention effect considering spatial effects; and 3) analysis of distance-based spillover and interaction with the intervention, characterizing the functional distance over which the spillover effect was present. R 3.4.2^17^ was used for all statistical analyses with the following packages.^18–31^

### Exploratory spatial analysis.

We explored spatial patterns in the intervention assignment through the use of a join count statistic,^32^ and spatial correlation of the outcome was assessed using Moran’s I.^33^ Spatial heterogeneity of the effect of bed nets over the study region was considered through the use of geographically weighted regression (GWR).^34^ Evidence of a spillover effect across cluster boundaries was assessed using a novel method we developed called cluster reallocation. The spatial patterns were also assessed visually through the use of maps. To the extent of our knowledge, with the exception of Moran’s I, this was the first time these methods have been applied to a CRT.

The join count statistic was calculated to assess whether there was a spatial pattern in treatment assignment. The join count method assesses spatial correlation for binary variables and involves counting all pairs of neighboring (Queen’s case) clusters in the trial by type of adjacency: intervention–intervention, control–control, or intervention–control.^32^ Neighbors were defined using Queen’s case where clusters that share a boundary or vertex were considered neighbors. Using a hypothesis testing framework, we then assessed whether the observed counts of these three possible adjacencies in the trial deviate from the expected counts based on a random pattern.^33^ Although the allocation of intervention to clusters is based on a random process, it could still result in a nonrandom spatial pattern. For example, randomization could result in all control clusters being in one area and all intervention clusters being in another area of the study. In this case, the study area could be split into two sections: an area with only intervention clusters present and an area with only control clusters present; this may present issues when trying to measure spatial effects as few intervention clusters may border control clusters.

Moran’s I statistic was used to assess the presence of global spatial autocorrelation in the SMRs at the cluster level. Moran’s I is an extension of Pearson’s product–moment correlation into two dimensions; it considers the strength of association and the spatial lag over which it is present. The SMR was calculated for each cluster, with a binary spatial weight matrix (Queen’s case) used to represent the connectivity between clusters. The spatial weight matrix takes a value of one if the clusters share a boundary or vertex, and zero otherwise. Moran’s I was calculated for the whole study area, and for the control and intervention clusters separately. Moran’s I was also calculated on Pearson's residuals from a multilevel model with a random effect (IID) for cluster and a fixed effect for intervention. The residuals were aggregated to the cluster level, which calculates Moran’s I adjusted for the intervention effect and the clustering.

*P*-values for Moran’s I were calculated using Monte Carlo simulation. This was achieved through a permutation test where the values for each cluster were shuffled to different locations and the statistics recalculated. This process was repeated many times and the observed value was compared with the sampling distribution of the simulated values to test for evidence of deviation from a random spatial pattern.

Spatial heterogeneity of the intervention estimates was explored using GWR.^34^ This method involved applying a regression model to a spatial subset of the data (a neighborhood) and then recording the coefficients of that model; a different neighborhood is then chosen and the model reapplied. This process was repeated over the entire study area to give one estimate of the coefficient for each neighborhood. As is standard, the neighborhood was a radius around each point. The distribution of the coefficients was then explored visually on a map, helping to determine sources of heterogeneity in the data. In situations where data are spatially heterogeneous, GWR produces coefficient estimates that vary over space, indicating local areas of departure from a global process. A Poisson regression model without a random effect for cluster was used for the GWR.

To assess for the presence of spatial spillover, we developed a new method called cluster reallocation, which considers how changes in the definition of cluster boundaries affect the intervention estimates of the trial. Cluster reallocation is a computationally intensive method that involves reallocating individuals to either the intervention or control arm based on their proximity to cluster boundaries. At each step, a model is applied to estimate the intervention effect. In this analysis, clusters were dilated (buffered) incrementally between 0 m (original case: no change to cluster) and 1,000 m in steps of 100 m. The process was carried out independently for intervention and control clusters.

At each 100 m increment, households were reassigned to intervention or control clusters, and the main trial model refitted. In the absence of spillover, we hypothesized that as the size of either control or intervention clusters grew, estimates of the intervention effect would attenuate to the null as differences between the intervention and control arms are diluted. However, if alternatively, a spatial spillover is present, then we would expect the magnitude of the intervention effect estimate to increase over the functional distance of the spillover, as the intervention cluster is dilated.

### Modeling spatial dependence.

The main result of the original trial article^3^ was replicated using a IID for cluster and a fixed effect for the intervention. Three approaches were considered for adjusting for spatial structure: a conditional autoregressive model (CAR)^35–37^; a Besag, Yorke, and Mollie model (BYM)^38^; and a Gaussian process model (GPm).^39,40^

The three spatial models were chosen for the different ways they incorporate spatial dependency. The approaches differ by whether they assume the underlying spatial process is discrete, called a Gaussian Markov random field (GMRF), or continuous, called a Gaussian process (GP). A GMRF is a collection of spatially indexed random variables with a Markov property, where all possible combinations of the random variables are multivariate normally distributed (MVN).^41^ Gaussian Markov random fields are commonly used when data are recorded for distinct areas covering an entire region, such as clusters in a CRT. A GP assumes that the spatial process is MVN, typically with a mean of zero and a covariance function that incorporates distance; therefore, changes in outcome due to the spatial process are a function of distance. Gaussian processes are commonly used when data are recorded for some points in an area and information is missing in other locations, such as households in a CRT.^42^ In classical spatial statistics, GMRFs refer to areal data and GPs to geostatistical models.^15,36^

The CAR and BYM models incorporate spatial structure at the cluster level. Fitting a CAR model to the data requires the aggregation of households to clusters as the model is restricted to only one observation per spatial area. By contrast, the BYM model can be fitted to the individual (household)-level data. The CAR model includes a spatially structured random effect for the clusters. The BYM model extends the CAR model to include an additional independent random effect and relaxes the link of one observation per cluster. The spatially structured random effect relaxes the assumption of independence between clusters and allows clusters that are adjacent to share information. A binary spatial weight matrix (Queens’s case) was used where clusters sharing a common boundary or vertex were considered to be adjacent. The BYM model effectively reproduces the standard approach to analyzing a CRT, but with an additional spatial effect at the cluster level.

The GPm incorporates spatial structure at an individual level (here: household). Gaussian process models tend to have dense spatial matrices, which makes computation difficult. Fortunately, a link between the continuously indexed GP and the discretely indexed GMRFs has been proposed, which uses stochastic partial differential equations (SPDEs).^43^ In short, Lindgren et al.^43^ demonstrated that a Matérn covariance model (a GP with a constant mean, and Matérn covariance) is a solution to an SPDE. They showed that approximating an area with a finite number of triangles or a “mesh” allows the solution of the SPDE to be represented as the weighted sum of the vertices of the “mesh.” Then assuming a Markov property on the mesh, it can be modeled as a GMRF. The GP is a solution of an SPDE, and the SPDE can be approximately solved by using a GMRF. Thus, the GP can be modeled using GMRF methods through the use of the mesh. The choice of mesh is a trade-off between how accurately the area can be represented and computational costs. Further adjustments can be made so that the mesh is finer in locations with data and less fine where there are less data (or information). Further details of the SPDE approach are described by Simpson et al. and Blangiardo et al.^44,45^

The four models in this section were fitted using integrated nested Laplace approximation (INLA), using noninformative priors.^25^ Integrated nested Laplace approximation is a deterministic algorithm which has proven to be capable of providing fast and reliable results for a wide range of models.^45,46^ Integrated nested Laplace approximation was well suited to this analysis because of the complexity of the model types and the size of data when accounting for spatial structure (12,000 observations giving a spatial weight matrix with 144 million elements). Once fitted, the posterior distributions of all models were sampled and the mean and 95% credible intervals (CrIs) calculated. Further details of the models are provided in Table 1.

**Table 1:** Estimate of intervention effect by spatial model type

Model	Standardized mortality ratio (95% credible interval) of intervention*	Equation	Description
IID	0.83 (0.71–0.98)	$y_{ijk}=\alpha +\beta x_{i}+u_{j}$	Multilevel model with independent random effect for cluster
Conditional autoregressive model	0.84 (0.72–0.98)	$y_{ijk}=\alpha +\beta x_{i}+v_{j}$	Conditional autoregressive model
BYM	0.84 (0.72–0.98)	$y_{ijk}=\alpha +\beta x_{i}+u_{j\;}+v_{j}$	Besag, Yorke, and Mollie model
GPm	0.83 (0.70–0.97)	$y_{ijk}=\alpha +\beta x_{i}+u_{j}+v_{k}$	Gaussian process model fitted using stochastic partial differential equation approach
IID + discordant distance	0.82 (0.70–0.95)	$y_{ijk}=\alpha +\beta x_{i}+u_{j}+\psi d_{k}$	Distance to discordant pair included in the model
BYM + discordant distance	0.84 (0.72–0.98)	$y_{ijk}=\alpha +\beta x_{i}+u_{j\;}+v_{j}+d\psi_{k}$	
GPm + discordant distance	0.82 (0.70–0.96)	$y_{ijk}=\alpha +\beta x_{i}+u_{j}+v_{k}+\psi d_{k}$	

Notation		
Observed variables	$y_{ijk}$	Outcome for the $k^{th}$participant in the $j^{th}$cluster in the $i^{th}$treatment arm
	$x_{i}$	Binary variable for intervention effect
	$d_{k}$	Distance to discordant pair
Parameters	α, β, ψ	Intercept, intervention effect, and spatial spillover effect
Random effects/unobserved variables	u	Independent and identically distributed random effect where $u∼N\left(0,\sigma^{2}\right)$
	$v_{j}$	Cluster level spatially correlated random effect where $v_{j}∼\;\mathrm{MVN}(0,\sigma^{2}\text{Σ}$) and $\text{Σ}={\left(i-\rho W\right)}^{-1}$
	$v_{k}$	Individual level spatially correlated random effect where $v_{k}∼\mathrm{MVN}(f\left(z\right),\;\sigma_{v}^{2})$ and f∼GP(μ(·)Σ(d))
Spatial	ρ, W	Spatial correlation parameter, spatial weight matrix
	μ(.), Σ(d)	Mean function, covariance function based on distance

BYM = Besag, Yorke, and Mollie model; GPm = Gaussian process model; IID = multi-level model with a random effect.

### Spillover effect and interaction.

A frequentist approach was used for exploring spillover and interaction as this is more commonly used in the analysis of CRTs.^47^ Evidence for positive spillover was assessed directly, by including distance to discordant pair as a variable in the IID model. The form of the distance variable was explored further to account for nonlinearity by using quadratic terms. In the geographic literature, this form of variable is often referred to as “distance decay.”^48^

Effect modification of the intervention effect by distance to discordant pair was assessed by including distance as a continuous variable using the IID model with an interaction term. For ease of interpretation, an interaction term with a binary distance variable (threshold 400 m) was used. The distance of 400 m was chosen as the spillover effect appears to attenuate at this distance based on the exploratory spatial analyses.

Robustness to distributional assumptions for the spillover effect was assessed using parametric bootstrapping.^49,50^ We calculated 2,000 bootstrap samples, and these were performed at the sciCORE scientific computing core facility, University of Basel (http://scicore.unibas.ch/).

Distance to discordant household was also fitted for the BYM and GP models (using INLA and, therefore, Bayesian inference) to see if accounting for spatial structure affected the estimate of the spillover effect of the intervention.

## RESULTS

### General characteristics.

There were 11,944 compounds, with 6,123 (51.2%) compounds receiving ITNs. Over the course of the trial, there were 861 deaths, resulting in an SMR of 1.24. The SMR in the control arm was 1.37 and was 0.25 units higher than that in the intervention arm (SMR 1.12). The median distance to the nearest discordant household was 618 m (interquartile range [IQR] 311–1,106 m). The median distance to the nearest bed net for control households was 591 m (IQR 305–1,010 m). The main trial results and a spatial analysis have been presented previously.^3,9^

### Exploratory spatial analysis.

There was no suggestion of a spatial pattern for the allocation of treatment for either the intervention or the control, as can be seen in Table 2 (join count statistic). There was strong evidence (*P* < 0.001) to suggest a spatial pattern for the SMR aggregated at the cluster level. This pattern was consistent when only considering the control clusters and when tested on residuals after adjusting for intervention, but was no longer apparent when only the intervention clusters were tested.

**Table 2:** Summary of analyses of spatial patterns of dependence, heterogeneity, and spillover

Method	Test statistic	*P*-value
Spatial correlation of intervention allocation		
Join count—control	10.105	0.979
Join count—intervention	11.282	0.744
Spatial correlation of SMR		
Moran’s I—entire study area	0.237	< 0.001
Moran’s I—control	0.396	< 0.001
Moran’s I—intervention	0.095	0.176
Moran’s I—residuals	0.134	0.0198
Spatial heterogeneity of intervention		SMR
Geographically weighted regression, median (interquartile range)	–	0.94 (0.77–1.09)
Impact of spillover on intervention effect*	–	SMR
Cluster reallocation method		
Original cluster definition	–	0.827
Controls cluster larger, mean (min, max)	–	0.885 (0.855–0.916)
Intervention clusters larger, mean (min, max)	–	0.781 (0.742–0.827)

SMR = standardized mortality ratio.

* This summary represents the mean of the intervention estimates that derive from increasing either the control or intervention cluster boundaries based on the cluster reallocation method.

The IQR for the GWR intervention estimate ranged from 0.77 to 1.09, suggesting some spatial heterogeneity and most areas having a beneficial intervention effect. Despite this, there were areas where the intervention estimate is greater than one. Of the 96 clusters in the trial, 37 (38.5%) had an intervention estimate greater than one and of these, 22 were control clusters. Figure 1 presents maps of the intervention assignment, spatial distribution of SMR at the cluster level, and the intervention estimates from GWR. There is no obvious pattern, with the effect of bed nets appearing unrelated to whether surrounding areas have high and low mortality ratios, or the density of intervention clusters nearby.

**Figure 2.**
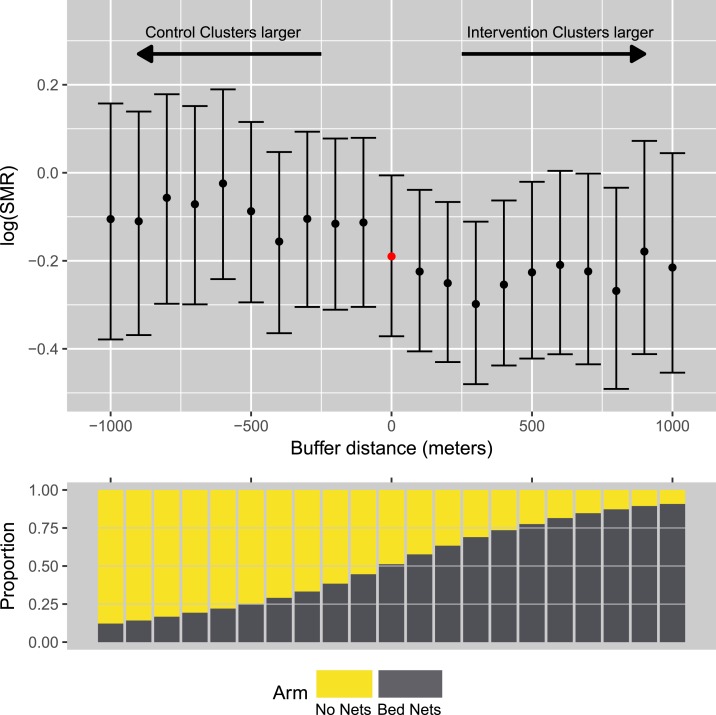
Change in effect estimate calculated by cluster reallocation of intervention participants to the control arm and vice versa. This figure appears in color at www.ajtmh.org.

The cluster reallocation method provided a strong suggestion of a spillover effect from intervention clusters toward control clusters. Expanding the intervention cluster boundary resulted in stronger (bed nets more effective) intervention estimates compared with the original cluster definitions up to around 400 m (Figure 2). This is consistent with individuals within 400 m of the intervention receiving an indirect benefit due to proximity. Expanding the control cluster attenuated the effect estimate to the null. This was consistent with an absence of spillover from control to intervention, as with each increase in buffer, the newly defined intervention and control arms contain more similar participants. Past 500 m, the number of participants in each arm became very unbalanced, and thus, the point estimates from these models should be treated with caution (Figure 2, lower graph). However, even when the buffering was at 1,000 m, the smaller arm still had greater than 1,000 observations, which was reflected in the consistent size of the CIs.

Analysis of the spatial autocorrelation of cluster-level SMRs, the heterogeneity in the intervention estimate over space, and the behavior of the intervention estimate under cluster dilution were all suggestive of a spatial spillover effect from the intervention to the control clusters. Furthermore, this effect was unlikely to be an artifact of the initial intervention allocation to the clusters, as there was no evidence of a spatial pattern in the cluster allocation.

### Spatial models.

Bed nets were associated with a 17% reduction in all-cause mortality for children aged 6–59 months. For the CAR and BYM models, adjusting for cluster-level spatial effects made negligible difference to estimated effects, and no difference to the conclusions of the trial. The GPm also resulted in near-identical estimates to that of the nonspatial model (Table 1).

Adjusting for distance to discordant pair did not greatly influence the estimate of the intervention effect, and this was consistent even when taking account of the spatial structure of the trial using the BYM and GP approaches.

### Spillover effect and interaction.

Distance to discordant pair was strongly associated with child mortality (*P* = 0.005); for every additional 100 m that a control household was from an intervention household (and vice versa), the mortality increased by 1.7% (SMR 1.017, 95% CI: 1.006–1.026). The result was consistent when adjusting for spatial structure and was robust to distributional assumptions with a bootstrapped estimate of 1.014 (95% CI: 1.005–1.025) (Table 3).

**Table 3:** Spillover effect of distance to discordant pair presented by model type and interaction of distance with bed net intervention

Variable	Model	SMR (95% credible interval)
Distance to discordant pair (per 100 m)	IID	1.017 (1.006 to 1.026)
	Besag, Yorke, and Mollie model	1.012 (1.004 to 1.020)
	Gaussian process	1.018 (1.005 to 1.029)
	Bootstrapped model (95% CI)	1.014 (1.005 to 1.025)
Stratum-specific SMRs	(Global test for interaction, *P* = 0.214)	SMR (95% CI)
Distance to discordant pair	** **Intervention	
400 m or nearer	** **Bed nets	1.00
	** **No bed nets	1.05 (0.79 to 1.40)
** **Further than 400 m	** **Bed nets	1.00
	** **No bed nets	1.29 (1.05 to 1.57)
Intervention	Distance to discordant pair	
** **Bed nets	** **Further than 400 m	1.00
	** **400 m or nearer	0.90 (0.72 to 1.13)
No bed nets	** **Further than 400 m	1.00
	** **400 m or nearer	0.74 (0.64 to 0.95)

SMR = standardized mortality ratio.

There was no statistical evidence (*P* = 0.214) for effect modification between the distance to discordant pair and use of bed nets. However, the study was not powered to test for interaction, and thus likely has low power.^51^ Contrastingly to the statistical evidence, the stratum-specific SMRs were suggestive of interaction. The distance to the nearest bed net household from a control household was associated with reduced mortality, with increasing distance resulting in increased mortality. By contrast, the distance to the nearest control household from a bed net household was not associated with mortality. Treating distance to discordant pair as binary (threshold of 400 m) suggests no increase in mortality for control households living within 400 m of bed net households, but did suggest an increase in mortality for those living further than 400 m away (Table 3). These results were consistent with the evidence of a spillover effect and the results in the original and spatial analysis of the Binka et al. trial.^3,9^

## DISCUSSION

We examined the existence of spatial spillovers and the impact of spatial effects on the overall trial conclusions in a CRT of ITNs. Multiple approaches strongly suggested evidence of a positive spatial spillover effect due to being near households who use bed nets. Allowing for detailed spatial correlations and spillover effects did not change the primary conclusions of the trial.^3^

Our analysis suggests that for every additional 100 m a control household was from an intervention household (and vice versa), the standardized child mortality ratio increased by 1.7% (SMR 1.017, 95% CrI: 1.006–1.026). Bed nets were associated with a 17% reduction in all-cause mortality for children aged 6–59 months (SMR 0.83, 95% CI: 0.71–0.98). This effect estimate remained robust to models with different spatial specifications and raises the question of whether standard CRT analyses are always robust to spatial spillover effects. This trial was conducted over a large study area, with large clusters, meaning that spillover effects may need to be substantial to impact the study results. An alternative explanation is that the spatial models were also subject to the same biases as the standard CRT model. Spatial effects of different strengths and distances could be tested through simulation studies to test the impact on CRT results.

Although there was no statistical evidence of interaction (*P* = 0.214), the stratum-specific effects suggested that distance from bed net households affects mortality, but distance from control households does not. Furthermore, it suggests that the use of bed nets was more effective when comparing individuals more than 400 m apart. This interaction effect is plausible; if spillover was present, we may expect control households to receive a benefit from being nearby to intervention households, but not vice versa. Moreover, we would expect the mortality of control households that were close to bed net households to be more similar to intervention households than those who were further away. The assessment of interaction between distance from bed nets and the use of bed nets should be evaluated in other existing ITN CRT data where locations have been collected. In addition, the interaction could be assessed in studies with more complex interventions which include additional vector controls.

We were surprised at the strength of the spillover effect, but using bootstrapping to test robustness to distributional assumptions of the model, we obtained consistent results (SMR 1.014, 95% CI: 1.007–1.027). The spillover effect could be explained by reduced populations of mosquitoes in areas near ITNs and was consistent with “mass killing” effects, found previously.^7,12^ Despite this, adjusting for distance to discordant household had negligible impact on the main trial result.

There were several limitations of this analysis; the data were aggregated at a household level, which may have resulted in potential loss of information. However, the results were consistent with an individual-level analysis and any spatial analyses would require aggregating the spatial information at a household level. There were also gaps between some of the clusters where no spatial data were collected, which may have affected results.

A further weakness is the use of household coordinates to represent the spatial structure or mechanism of the spillover. Household location was at best one of the many locations that an individual visited during the study period. This resulted in omissions of many of the spatial locations related to each participant. However, this was probably of minimal impact in the context of mosquitoes and ITNs where transmission is likely to happen at night when at home. Despite this, the value of household location in other contexts should be considered. In future, the possibility of tracking movement of people or mosquitoes may provide improved insights into the mechanism behind spatial spillover effects.

These analyses demonstrate that collection of GPS data allows exploration of intervention mechanisms beyond the creation of maps. The spatial analyses helped identify the distance over which spillover effects were present. They also reflected the spatial heterogeneity of the effect estimate, and potential effect modification of the intervention effect by distance. In this context, it demonstrated that individuals living within 400 m of an ITN may receive an indirect benefit from the intervention. This could have important implications for implementation of ITNs in scarce resource settings where the number of available nets may be fewer than the number of individuals who require them.

A key benefit of our approach was that it did not require any additional data collection as the spatial analysis only needs GPS coordinates which are often collected in CRTs. Therefore, this approach could be used to reanalyze previous geographical CRTs that collected coordinates, thus gaining extra utility from previously collected data. We explored a range of spatial methods, allowing for comparison of different spatial approaches, and the conclusions of our analysis remained consistent with the original analyses conducted in the 1990s.

A further difficulty of spatial analyses is that they require specialist spatial statistical skills and would be difficult to conduct without considerable effort or further training for a trial statistician. The exploratory approaches require familiarity with spatial data, although the actual analyses were relatively straightforward once the data were loaded. By contrast, the spatial models fitted using INLA require much greater effort, and furthermore, the GPm is very computationally intensive and may be less feasible on very large trials.

These analyses add to the expanding literature on the spatial indirect effects of ITNs on mosquitoes and spatial analyses of CRTs.^6,10^ Further details of other approaches can be found in a review of methods used for spatial analysis of CRTs published in 2017.^10^ The review categorized the approaches into spatial variable and spatial modeling approaches.

This analysis raises important implications for the design of future trials. Consideration of the spatial structure of the trial design and the possibility of spillover effects is needed. Alternative design approaches have been suggested to reduce or help measure spillover effects. Silkey et al. propose the use of stepped-wedge trials. McCann et al. developed an approach that involves fully including or fully excluding available clusters in a defined study region.^52,53^ Combining the design approaches with historical spatial analyses of existing trial data could help provide a rich resource of information for decision makers to use when designing future studies.

In summary, despite evidence of a spatial spillover effect, the conclusions of the trial were unaffected by spatial model specifications. Use of ITNs was clearly beneficial for individuals, and there was compelling evidence that they provide an indirect benefit to individuals living nearby. Although this article demonstrates robustness of CRT analyses to spatial effects, this was the only scenario, and the clusters may have been large compared with the level of spillover in this study. Simulation studies could be used to evaluate the robustness of intervention estimates in CRTs for differing distances and strengths of spatial effects.
